# Supplementary material for: Transcriptome Analysis Reveals Vimentin-Induced Disruption of Cell–Cell Associations Augments Breast Cancer Cell Migration
Source: Cells. 2022 Dec 13;11(24):4035. doi: 10.3390/cells11244035 (PMC9776984; doi:10.3390/cells11244035)
Supplement: Supplementary file 1 [file cells-11-04035-s001.zip › DEGs and enrichment analysis.zip!/DEGs and enrichment analysis/WT_VIMvsVector GO enrichment/ALL/WT_VIMvsVector_all.MFbar.pdf]

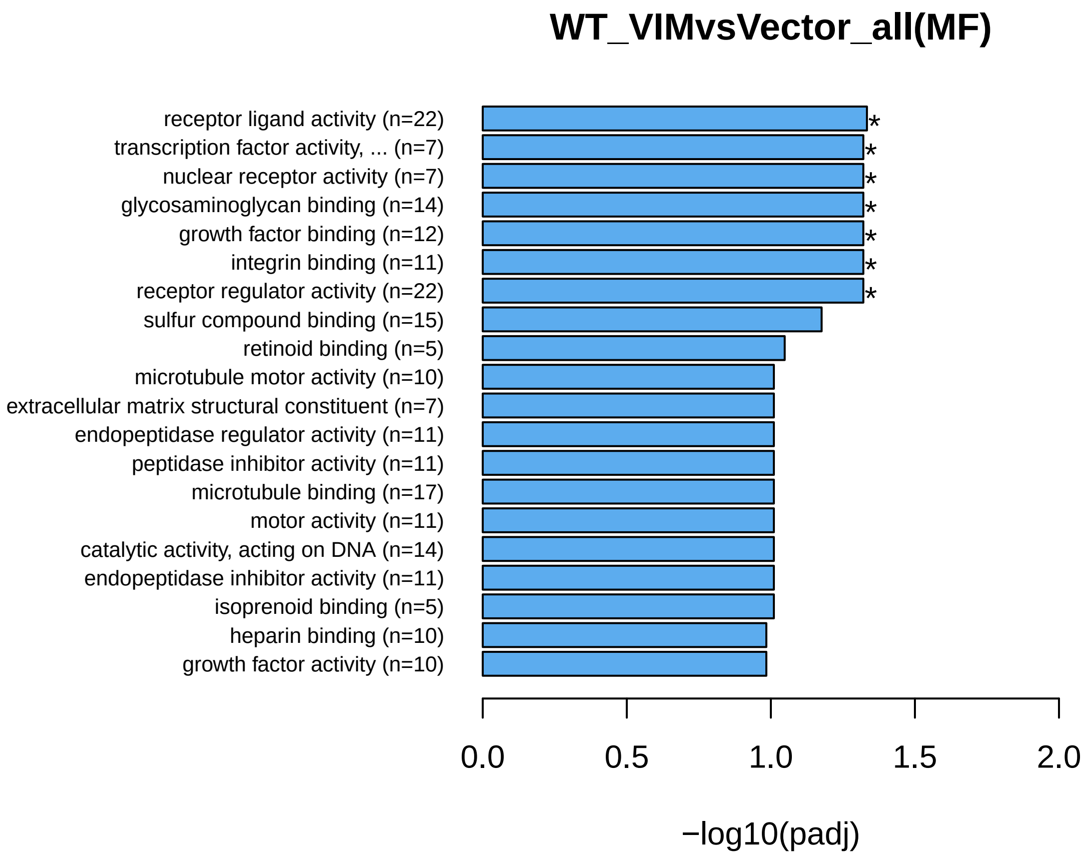
WT_VIMvsVector_all(MF)
receptor ligand activity (n=22)
transcription factor activity, ... (n=7)
nuclear receptor activity (n=7)
glycosaminoglycan binding (n=14)
growth factor binding (n=12)
integrin binding (n=11)
receptor regulator activity (n=22)
sulfur compound binding (n=15)
retinoid binding (n=5)
microtubule motor activity (n=10)
extracellular matrix structural constituent (n=7)
endopeptidase regulator activity (n=11)
peptidase inhibitor activity (n=11)
microtubule binding (n=17)
motor activity (n=11)
catalytic activity, acting on DNA (n=14)
endopeptidase inhibitor activity (n=11)
isoprenoid binding (n=5)
heparin binding (n=10)
growth factor activity (n=10)
*
0.0
0.5
1.0
1.5
2.0
−log10(padj)
